# To compare the efficacy of two kinds of Zhizhu pills in the treatment of functional dyspepsia of spleen-deficiency and qi-stagnation syndrome:a randomized group sequential comparative trial

**DOI:** 10.1186/1471-230X-11-81

**Published:** 2011-07-15

**Authors:** Hongli Wu, Zhiwei Jing, Xudong Tang, Xinyue Wang, Shengsheng Zhang, Yanan Yu, Zhong Wang, Hongxin Cao, Luqi Huang, Youhua Yu, Yongyan Wang

**Affiliations:** 1 Institute of Basic Research in Clinical Medicine, China Academy of Chinese Medical Sciences, No. 16 Nanxiaojie, Dongzhimen nei, Beijing, 100700, China; 2 Xiyuan Hospital of China Academy of Chinese Medical Sciences, No.1 Xiyuan playground, Haidian District, Beijing 100091, China; 3 Dongzhimen Hospital affiliated to Beijing University of Chinese Medicine, No.5 Haiyuncang, Dongcheng District, Beijing 100700, China; 4 Beijing Traditional Chinese Medicine Hospital, Capital Medical University, No.5 Meishuguan Back Street, Dongcheng District, Beijing 100010, China; 5 China Academy of Chinese Medical Sciences, No. 16 Nanxiaojie, Dongzhimen nei, Beijing, 100700, China; 6 Institute of Chinese Material Medica, China Academy of Chinese Medical Sciences, No. 16 Nanxiaojie, Dongzhimen nei, Beijing, 100700, China; 7 Experimental Research Center, China Academy of Chinese Medical Sciences, No. 16 Nanxiaojie, Dongzhimen nei, Beijing, 100700, China

## Abstract

**Background:**

In Traditional Chinese Medicine (TCM) theory, functional dyspepsia (FD) can be divided into different syndromes according to different clinical symptoms and signs, and the most common one is spleen-deficiency and qi-stagnation syndrome that can be treated by Chinese traditional patent medicine ---- two kinds of Zhizhu pills, between which the primary difference in ingredients is that one contains immature orange fruit of Citrus aurantium L.(IFCA) and the other contains that of Citrus sinensis Osbeck (IFCS). The trial's objective was to compare the efficacy of two kinds of Zhizhu pills on symptom changes in patients with FD of spleen-deficiency and qi-stagnation syndrome.

**Methods:**

A randomized, group sequential, double-blinded, multicenter trial was conducted in patients with FD of spleen-deficiency and qi-stagnation syndrome at 3 hospitals in Beijing between June 2003 and May 2005. Participants were randomly allocated into two groups (IFCA group and IFCS group) in a 1:1 ratio, and respectively took one of the two kinds of Zhizhu pills orally, 6 g each time, 3 times a day, for 4 weeks. Statistical analysis was performed with use of a group sequential method, the triangular test (TT).

**Results:**

A total of 163 patients were randomized, and 3 patients were excluded from analysis because of early dropouts, leaving 160 patients (IFCA group: n = 82; IFCS group: n = 78) for statistical analysis. Three interim analyses were done after 62, 116, and 160 patients had completed their 4-week treatment, respectively. At the third interim analysis, the sample path crossed the upper boundary and the trial was stopped, the cure-markedly effective rates were 45% for IFCS group and 67% for IFCA group, respectively, the one-sided *p*-value was 0.0036, the median unbiased estimate of the odds ratio (OR) for the benefit of IFCA relative to IFCS was 2.91 with 95%CI: 1.40 to 6.06.

No adverse events were observed in the two groups.

**Conclusions:**

Zhizhu pills containing IFCA was superior to Zhizhu pills containing IFCS in the treatment of FD of spleen-deficiency and qi-stagnation syndrome. The application of group sequential analysis in clinical trials of TCM may offer some financial and ethical benefits.

**Trial Registration:**

Chinese Clinical Trial Registry (ChiCTR): ChiCTR-TRC-00000485

## Background

According to the proposition of an international committee meeting in Rome in 1991, the term "dyspepsia" refers to pain or discomfort centered in the upper abdomen [1], while discomfort refers to a subjective negative (or aversive) feeling, such as early satiety, fullness, bloating and nausea. In Rome I and Rome II reports [2,3], functional dyspepsia (FD) is defined as a persistent or recurrent dyspepsia for at least 12 weeks in the preceding 12 months, if there is no evidence for organic disease (including at upper endoscopy) that could cause the symptoms. An epidemiological survey of western countries showed that the prevalence of FD ranged from 11.5% to 14.7% [4]. It is also a common clinical condition in China, and the report on the incidence of FD in citizens of Tianjin, China, revealed that the proportion of patients with FD reached 23.29% of the total population [5].

In western medicine, the treatment of FD remains a major unsolved problem in gastroenterology, and this is a discouraging and unsatisfactory situation for treating physicians.

In traditional Chinese medicine (TCM), FD is considered nearly equivalent to the TCM term "stuffiness and fullness" [6], which is divided into different syndromes according to different clinical symptoms and signs. The basic syndromes include liver-stomach disharmony syndrome (qi-stagnation syndrome), fluid and food retention syndrome, dampness-heat of spleen and stomach syndrome, spleen-stomach weakness syndrome (spleen-deficiency syndrome), and cold and heat in complexity syndrome. It should be noted that in clinical practice, qi-stagnation syndrome and spleen-deficiency syndrome often come together, which is known as the spleen-deficiency and qi-stagnation syndrome and can be treated by Chinese traditional patent medicine ---- two kinds of Zhizhu pills [7]. Both of them are made from Rhizoma Atractylodis Macrocephalae (stir-baked) and Fructus Aurantii Immaturus (immature orange fruit, stir-baked), while their primary difference in ingredients is that one contains immature orange fruit of Citrus aurantium L.(IFCA) and the other contains that of Citrus sinensis Osbeck (IFCS). In clinical practice, both Zhizhu pills have been considered to be effective in the treatment of functional dyspepsia (FD) of spleen-deficiency and qi-stagnation syndrome. The trial's objective was to compare the efficacy of two kinds of Zhizhu pills on symptom changes in patients with FD of spleen-deficiency and qi-stagnation syndrome.

## Methods

### Trial design

A randomized, group sequential, double-blinded, multicenter trial was conducted in patients with FD of spleen-deficiency and qi-stagnation syndrome at 3 hospitals in Beijing between June 2003 and May 2005.

### Participants

Based on the Rome-II criteria and the Guiding principle for clinical research on new drugs of traditional Chinese medicine (trial implementation) [6], patients with FD of spleen-deficiency and qi-stagnation syndrome were enrolled in the study. The spleen-deficiency and qi-stagnation syndrome is defined as having the main symptoms and at least two of the accompanying symptoms, as well as pale tongue with whitish tongue coating and deep and thready pulse. The main symptoms include epigastric stuffiness and fullness, and asthenia. While the accompanying symptoms include epigastric stuffiness and fullness aggravated after meal, epigastric pain, decreased appetite, belching and acid regurgitation, fullness and discomfort in chest and hypochondrium, nausea and vomiting, and constipation or loose stool. Other inclusion criteria included TCM syndrome integral ≥8, aged 18 to 65 years old, and able and willing to provide a signed and dated informed consent form.

Patients who had gastric ulcer or duodenal ulcer revealed by upper gastrointestinal endoscopy, gastroesophageal reflux disease with typical symptoms like heartburn or acid regurgitation, or any malignant diseases were excluded from this study. Other exclusion criteria included pregnant or breast feeding women as well as patients with serious hepatic, cardiovascular, renal, or hematological diseases. Patients with a known history of hypersensitivity to Zhizhu pills were also excluded.

### Interventions

Both kinds of Zhizhu pills (Approval No. granted by State Food and Drug Administration of China (SFDA): 00406105), with a same instruction on usage and dosage, were provided by the experimental pharmaceutical factory, a subsidiary to China Academy of Chinese Medical Sciences (CACMS). The pills should be taken 6 g orally each time, 3 times a day after meals, for 4 weeks.

In addition to the above treatment, patients should not receive any concomitant medications associated with the treatment of this disease, and were also required to stop taking such drugs at least 1 week before their study entry, which include but not limited to anti-cholinergic drugs, antispasmodic agents, emetic agents, H_2 _receptor antagonists, and any other gastric motility drugs. Moreover, patients were also asked to stop smoking, drinking alcohol or tea throughout the trial.

### Outcomes

#### Primary outcome

We assessed each patient's symptoms before and after treatment by means of a rating scale (See Additional file 1), which was completed by the treating physicians when they interviewed their patients before and after treatment (at baseline and week 4). The rating scale consisted of 11 items (2 for the main symptoms, 7 for the accompanying symptoms and 2 for tongue and pulse) with 2 (yes or no) or 4 options (absent, mild, moderate or severe) for each item. Each option was represented by a fixed score, the higher the score, the more severe the symptom, and vice versa. The total score of the rating scale was called syndrome integral.

Based on the efficacy standards for patients with FD of spleen-deficiency and qi-stagnation syndrome recommended in the "Guiding principle for clinical research on new drugs of traditional Chinese medicine (trial implementation)" [6], the overall efficacy was judged as clinical recovery (the main symptoms and signs disappeared or almost disappeared, and therapeutic index ≥ 95%, which was defined as (1- after-treatment syndrome integral/before-treatment syndrome integral) ×100%, significant improvement (the main symptoms and signs improved significantly, and 70% ≤ therapeutic index < 95%), somewhat improvement (the main symptoms and signs somewhat improved, and 30% ≤ therapeutic index <70%), no change (therapeutic index < 30%), somewhat worsening, and significant worsening, etc.

For statistical convenience, the overall efficacy was categorized into 2 groups in this study. Clinical recovery and significant improvement were judged as cured and markedly improved, the cure-markedly effective rate, the primary outcome of this trial, was defined as the proportion of patients achieving clinical recovery or significant improvement; while somewhat improvement, no change, somewhat worsening and significant worsening were judged as not markedly improved.

#### Secondary outcome

The secondary outcome, gastric emptying rate, was affected by the group sequential stopping rules. Therefore, a conventional analysis of the secondary endpoints was not planned in this study.

### Randomisation and Allocation

A central-randomization scheme was performed in the study. The random allocation sequence was generated and conserved by the trial organizer, and participants were randomly allocated into two groups (IFCA group and IFCS group) in a 1:1 ratio.

### Blinding

This study was a double-blind clinical trial, with the patients, the treating physicians, the statisticians, the monitors and any other site personnel being unaware of group allocation. Efforts to maintain blinding included identical appearance, packaging and labeling of two kinds of Zhizhu pills.

Unblinding should be done by the statisticians when the data collection process was completed or the treating physicians when serious adverse events occurred.

### Sample size

Before the trial was initiated, the sample size was calculated according to the following hypotheses: based on the results of our previous study, we anticipated that the cure-markedly effective rates were 40% for IFCS group and 55% for IFCA group, respectively, and the type I and II error rates were chosen at their usual values of 0.05 (one-sided). Under these conditions, the required sample size with the use of a single-stage design and a one-sided test would have been 472 in total. Considering the recruitment- related difficulties and ethical problems, we designed this study with the triangular test (TT).

### Statistical methods

The triangular test was designed to test *H*_0_: log odds ratio θ = 0 with a (one-sided) type I error rate of α = 0.05 and a power of 1-β = 0.95 for θ_R _= 0.6061. The interim analyses were planned approximately after every 50 patients completed their 4-week treatment.

In brief, in the triangular test [8,9], two straight lines, called the boundaries of the test, delineate a continuation region (between these two lines), and the equations of the boundaries are U: B = 6.5761+0.1515 V for the upper boundary and L: B = -6.5761+0.4546 V for the lower boundary.

The consecutive points (V, B), obtained from the data collected at each analysis, define a sample path from the left to the right of the continuation region. The trial would be stopped to conclude that the IFCA group was superior to the IFCS group if the sample path crossed the upper boundary, stopped without that conclusion if the sample path crossed the lower boundary, and continued otherwise. However, the definitive judgment of whether to conclude that the IFCA group was superior to the IFCS group could be based on whether the one-sided p-value satisfied p ≤ 0.05.

## Results

### Study population

A total of 163 participants were included in the study, of whom 3 (1 in the IFCA group and 2 in the IFCS group) dropped out prematurely for personal reasons. The remaining 160 participants were evaluable in the statistical analysis. All the results presented refer to this population.

### Participant flow

**Figure 1:**
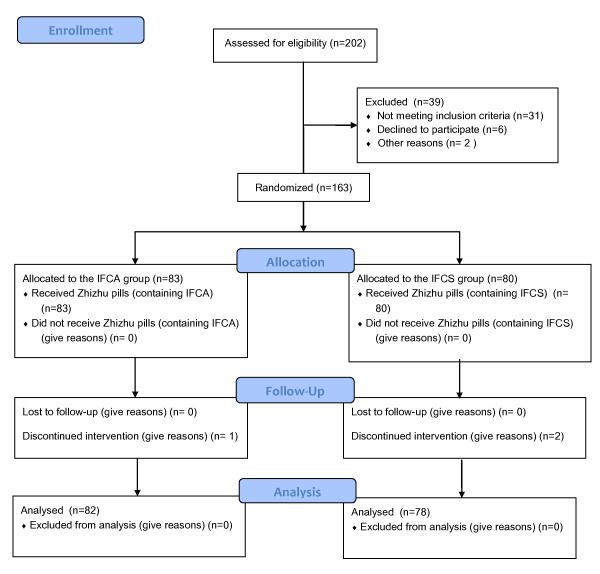
**The flow of the participants in the study**.

The flow of the participants in the study is summarized in Figure 1.

### Baseline data

Table 1 shows the general characteristics of evaluable population. No significant differences were identified between the two groups for any of these parameters, such as gender, age, course of disease and before-treatment syndrome integral.

**Table 1:** Baseline patient characteristics

Characteristic	IFCS(n = 78)	IFCA(n = 82)
Gender(male/female)	22/56	31/51
Age(years)	47.2 ± 13.3	48.6 ± 14.5
Course of disease(months)	19.6 ± 20.5	17.4 ± 21.6
Before-treatment syndrome integral	17.3 ± 4.8	17.0 ± 4.3

### Primary outcome (sequential analysis)

**Figure 2:**
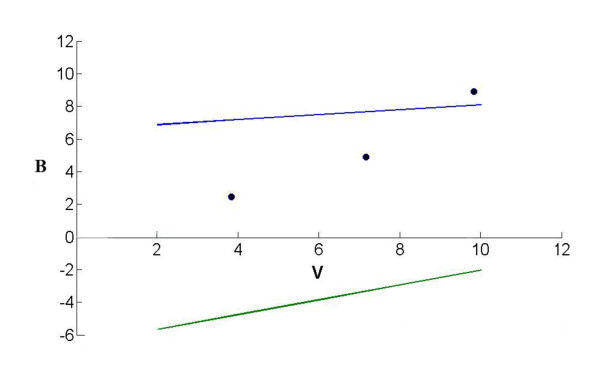
**Design of the triangular test (upper boundary, B = 6.5761+0.1515 V; lower boundary, B = -6.5761+0.4546 V) and the results of the three sequential analyses for the comparison of two kinds of Zhizhu pills**. On the third analysis, the sample path crossing the upper boundary indicates the Zhizhu pill containing IFCA is superior.

Table 2 shows the cumulated numbers of evaluable population at each step and the corresponding values of the two statistics V and B computed for each sequential analysis. Figure 2 represents the triangular test and the corresponding sample path.

**Table 2:** Cumulative results for the triangular test applied to the trial in FD of spleen-deficiency and qi-stagnation syndrome

Analysis	**n**_**IFCA**_	**n**_**IFCS**_	**S**_**IFCA**_	**F**_**IFCA**_	**S**_**IFCS**_	**F**_**ISCS**_	B	V
1	32	30	20	12	14	16	2.45	3.83
2	60	56	38	22	26	30	4.89	7.16
3	82	78	55	27	35	43	8.89	9.82

Three interim analyses were done after 62, 116, and 160 patients had completed their 4-week treatment, respectively. At the third interim analysis, the sample path crossed the upper boundary and the trial was stopped. At stopping, the after-treatment syndrome integral were 5.5 ± 2.1 for IFCA group and 6.3 ± 2.3 for IFCS group, the cure-markedly effective rates were 67% for IFCA group and 45% for IFCS group, the one-sided *p*-value was 0.0036, the median unbiased estimate of the odds ratio (OR) for the benefit of IFCA relative to IFCS was 2.91 with 95%CI: 1.40 to 6.06.

### Adverse events and tolerance

Throughout the trial, the two kinds of Zhizhu pills were generally safe and well-tolerated by all patients. No major adverse events were observed in the two groups.

## Discussion

During the past two decades, the pathophysiology of FD has been under investigation, and multiple mechanisms such as abnormal gastric emptying, visceral hypersensitivity, impaired gastric accommodation and central nervous system factors are likely involved. So the possibilities of pharmacological therapy for FD are still limited, however, experience of using prokinetics, tricyclic antidepressants, selective serotonin-reuptake inhibitors (SSRIs), proton-pump inhibitors (PPIs), and several alternative techniques (such as acupuncture, multicomponent herbal medications and hypnotherapy) has been accumulated [10]. Although the efficacy of some established treatments (e.g. antisecretory agents or prokinetics) has been proven in placebo-controlled trials, the majority of these trials have shown only minor advantages of these drugs compared with placebo [11,12], spontaneous improvement may partially explain at least part of the placebo response [13].

In many countries, some physicians use various herbal medications for the treatment of FD, and their clinical experience appears to support the use of the treatments, but the efficacy of these treatments has been lacking the supporting of randomized controlled studies in the past decades. Recently, several well-designed placebo-controlled clinical trials have provided evidence for the efficacy of herbal medications used in the treatment of dyspepsia [14].

Based on the concept of TCM, two kinds of Zhizhu pills can be used to treat FD of spleen-deficiency and qi-stagnation syndrome. But the efficacy of most herbal medications is influenced by many factors which may lead to unstable efficacy in clinical practice. These factors include the differences in the variety of Chinese medicinal herbs, growing areas, harvesting seasons and the processing and preparing of these herbs, etc., of which the variety is a most influential factor. Thus, it is of great importance to make a comparison of a same prescription made from different varieties of a medicinal herb so as to further improve its clinical efficacy.

Pharmacological experiments [15] have indicated that the bioavailability of Zhizhu pills containing IFCA was superior to those containing IFCS, because flavonoid compounds such as naringin and hesperidin, which can be converted to naringenin and hesperitin in the body and affect the gastrointestinal motility, are part of the substance basis for the efficacy of Zhizhu pills in treating abdominal distension, and the pharmacokinetic parameters after the administration of two kinds of Zhizhu pills have shown that the sum AUC of naringenin and hesperitin of Zhizhu pills containing IFCA was much bigger than that of Zhizhu pills containing IFCS, which indicated that the efficacy of the former was superior to the latter, this conclusion was consistent with the findings of our study.

As for the methodological aspects of our study, if the classic single-stage design (SSD) was applied, the sample size needed would have been 472 patients. But given the difficulties encountered in recruitment of eligible patients, it was most likely that it would not have been possible, in only three centers, to bring this study to its end within three years. Moreover, since the primary outcome could be obtained relatively quickly when compared with the recruitment rate, this trial was planned with a group sequential design.

In group sequential designs, the sample size is a random variable whose distribution depends on the true treatment difference and on the stopping rule used. For this trial, triangular test (TT) was considered to be appropriate, the TT is a special case of a class of sequential designs developed by Andersonthis [16]and advocated by Whitehead [8,17]. Veronique et al. [18] indicated that the TT, of the sequential tests, seems to be the most appealing with regard to statistical properties: type I and II errors are correctly maintained to their desired values, and it offers a substantial decrease in sample size compared not only with single-stage design (SSD) but also with most of the other tests, whatever the frequency of the analyses. Simulation studies indicated that [19] the use of a TT-based sequential analysis design brought about a 50% reduction of the average sample needed, when compared to the fixed-sample design. In this study, using the TT-based sequential analysis, we saved 66% of the patient sample size. Moreover, patient recruitment in the study could be stopped shortly after 20 months from the recruitment of the first patient, which saved much time since the recruitment were initially planned about a five year period. This study also complied with ethical requirements and could make a case for wider use of group sequential designs in TCM clinical trials.

The statistical hypotheses made in the planning phase were drawn from our previous experience. At the third analysis, the upper boundary was crossed, allowing the trial to be stopped, and the computations [9] provided the one-sided p-value which was just the one-sided type I error of this design, the resulting value was p = 0.0036, so we can conclude that Zhizhu pills containing IFCA was superior to Zhizhu pills containing IFCS. There are some discrepancies in the literature [20] about the cure-markedly effective rate of Zhizhu pills in treatment of FD. In our opinion, differences in the diagnosis of the syndrome and the definition of the cure-markedly effective rate may be possible explanations.

The major advantage of group sequential designs lies in the early stopping. However, the decision to stop a study early involves ethical, administrative, economic and not just statistical consideration. Statistics should be considered as a tool, which could trigger the procedure to stop a trial but statistics cannot supplant a trial safety committee.

Our study inevitably had its limitations. The trial, ended in May 2005, adopted the Rome II criteria, which was modified in 2006 (the Rome III criteria). The Rome I and II criteria did not account for meal-related symptoms and this was the fundamental change in Rome III criteria [21]. The other limitation of the present study was the lack of a treatment arm using placebo. In order to further investigate the efficacy of Zhizhu pills containing IFCA in the treatment of FD, a randomized placebo-controlled clinical trial is required.

## Conclusions

Zhizhu pills containing IFCA was superior to Zhizhu pills containing IFCS in the treatment of FD of spleen-deficiency and qi-stagnation syndrome. And the triangular test was successfully applied to this study, and offered some financial and ethical benefits. Thus, for future TCM clinical trials, a group sequential design should remain a strong consideration.

## Competing interests

The authors declare that they have no competing interests.

## Authors' contributions

CHX, WZ, JZW, TXD, WXY, ZSS and WHL contributed to the conception and design of the study. WZ and WHL drafted the manuscript. All authors contributed to the further writing of the manuscript. All authors read and approved the final manuscript.

## Pre-publication history

The pre-publication history for this paper can be accessed here:

http://www.biomedcentral.com/1471-230X/11/81/prepub

## Supplementary Material

Additional file 1 **The Rating Scale for FD of Spleen-deficiency and Qi-stagnation Syndrome**. The rating scale consisted of 11 items with 2 or 4 options for each item. Each option was represented by a fixed score. The Before-treatment score and After-treatment score were assessed at the 0th and (28 ± 2)th day of the trial respectively. Total score was called syndrome integral. Click here for file
