# Supplementary material for: To compare the efficacy of two kinds of Zhizhu pills in the treatment of functional dyspepsia of spleen-deficiency and qi-stagnation syndrome:a randomized group sequential comparative trial
Source: BMC Gastroenterol. 2011 Jul 15;11:81. doi: 10.1186/1471-230X-11-81 (PMC3160401; doi:10.1186/1471-230X-11-81)
Supplement: Additional file 1 — The Rating Scale for FD of Spleen-deficiency and Qi-stagnation Syndrome. The rating scale consisted of 11 items with 2 or 4 options for each item. Each option was represented by a fixed score. The Before-treatment score and After-treatment score were assessed at the 0th and (28 ± 2)th day of the trial respectively. Total score was called syndrome integral. [file 1471-230X-11-81-S1.DOC]

**The Rating Scale for FD**

**of Spleen-deficiency and Qi-stagnation Syndrome**

***Patient identification number__________ Patient’s Name_____***

[***Gender***](dict://key.0895DFE8DB67F9409DB285590D870EDD/patient gender) ***_____ Age_____***

***Date of first assessment___________________________***

***Date of second assessment___________________________***

**To rate the severity of the symptoms in patients who were diagnosed as FD of spleen-deficiency and qi-stagnation syndrome, administer this questionnaire. The higher the score, the more severe the symptoms.**

**Choose the best answer for each blank (only one answer** per blank).

| [**Clinical symptoms**](dict://key.0895DFE8DB67F9409DB285590D870EDD/clinical symptoms) **and signs** | **Score standards** | **Before-treatment score** | **After-treatment score** |
| --- | --- | --- | --- |
| **1****. epigastric stuffiness and fullness** | **0= absent**  **2= mild, rarely occur (in average equal to or less than 3 times a week), daily activities being unaffected**  **4= tolerable, sometimes occur ( in more than 3 times a week, but equal to or less than once a day), daily activities being somewhat affected**  **6= intolerable, frequently occur (in average more than once a day), daily activities being severely affected** |  |  |
| **2. asthenia** | **0= absent**  **2= mild, can manage some light physical activities**  **4= moderate, having difficulty with routine physical activities**  **6= severe, do not want to take part in any physical activities all the day** |  |  |
| **3. epigastric stuffiness and fullness**  **aggravated after meal** | **0= absent**  **1= mild, disappear within an hour after meal**  **2= moderate, last for 1-2 hours after meal**  **3= severe, last for at least 2 hours after meal** |  |  |
| **4. epigastric pain** | **0= absent**  **1= mild, rarely occur, daily activities being unaffected**  **2= tolerable, sometimes occur, daily activities being somewhat affected**  **3= intolerable, frequently occur, daily activities being severely affected, and analgesics required** |  |  |
| **5. decreased appetite** | **0= absent**  **1= reduction in food consumption equal to or less than** 1/4  **2= reduction in food consumption greater than 1/4 but equal to or less than 1/2**  **3= reduction in food consumption** greater than 1/2 |  |  |
| **6. belching and acid regurgitation** | **0= absent**  **1=belching rarely occur**  **2= belching sometimes occur and aggravated after meal**  **3=belching frequently occur and aggravated after meal, even with acid or food regurgitation** |  |  |
| **7. fullness and discomfort in chest and hypochondrium** | **0= absent**  **1= mild, rarely occur, daily activities being unaffected**  **2= tolerable, sometimes occur, daily activities being somewhat affected**  **3= intolerable, frequently occur, daily activities being severely affected** |  |  |
| **8.** [**nausea and vomiting**](javascript:showjdsw('showjd_0','j_0')) | **0= absent**  **1= rarely feel nausea**  **2= sometimes feel nausea and rarely vomit**  **3= frequently fell nausea and sometimes vomit** |  |  |
| **9a. loose stool** | **0= absent**  **1= stool not well-formed**  **2= loose stool, less than 4 times a day**  **3= very loose stool, equal to or more than 4 times a day** |  |  |
| **9b. constipation** | **0= absent**  **1= mild**  **2= moderate, having difficult with defecation**  **3= severe, purgatives required** |  |  |
| **10a. pale tongue** | **0=no**  **1=yes** |  |  |
| **10b. whitish tongue coating** | **0=no**  **1=yes** |  |  |
| **11. Deep and thready pulse** | **0=no**  **1=yes** |  |  |
| **Total score** | **­­­­** |  |  |

**Research Center: *____________________***

[**Investigator**](dict://key.0895DFE8DB67F9409DB285590D870EDD/investigator)**: *____________________***
